# Fractional flow reserve-guided coronary angioplasty using paclitaxel-coated balloons without stent implantation: feasibility, safety and 6-month results by angiography and optical coherence tomography

**DOI:** 10.1007/s00392-016-1019-4

**Published:** 2016-07-05

**Authors:** Tudor C. Poerner, Corinna Duderstadt, Björn Goebel, Daniel Kretzschmar, Hans R. Figulla, Sylvia Otto

**Affiliations:** Division of Cardiology, 1st Department of Medicine, University Hospital of Jena, 07747 Jena, Germany

**Keywords:** Optical coherence tomography, Drug-eluting balloon, Drug-coated balloon, PCI

## Abstract

**Background:**

Percutaneous coronary interventions (PCI) with drug-coated balloons (DCB) might be a promising trade-off between balloon angioplasty and drug-eluting stents, since DCB inhibit neointimal proliferation and limit duration of dual antiplatelet therapy. We investigated the safety, feasibility, and 6-month results of fractional flow reserve (FFR)-guided use of the paclitaxel-coated SeQuent Please^®^ balloon without stenting for elective PCI of de novo lesions.

**Methods and results:**

In 46 patients (54 lesions) with stable symptomatic coronary artery disease (CAD), a FFR-guided POBA (plain old balloon angioplasty) was performed. In case of a sufficient POBA result with residual stenosis < 40 %, FFR > 0.8 and no severe dissection, the target lesion was finally dilated using the DCB. Quantitative coronary angiography (QCA) was performed before and after the index procedure and at 6-month follow-up (f/u) to calculate late lumen loss (LLL) and net luminal gain (NLG). Optical coherence tomography (OCT) was performed at f/u to assess vascular remodeling. DCB-only treatment was applied to 43 patients (51 lesions), while 3 patients (3 lesions) needed provisional stenting. Invasive f/u was completed in 39 patients (47 lesions). At the stenotic site, the lumen diameter showed a trend toward progressive increase at f/u (LLL: −0.13 ± 0.44 mm, n.s.; NLG: 1.10 ± 0.53 mm, *p* < 0.001) without aneurysm formation or restenosis after DCB-only treatment.

**Conclusions:**

FFR-guided DCB-only PCI of de novo lesions appeared feasible and safe in stable CAD with clopidogrel discontinuation after 4 weeks, showing a trend toward positive vessel remodeling without lumen loss at 6 months.

Clinical trial registration http://www.clinicaltrials.gov. Unique identifier: NCT02120859

**Electronic supplementary material:**

The online version of this article (doi:10.1007/s00392-016-1019-4) contains supplementary material, which is available to authorized users.

## Introduction

Plain-old balloon angioplasty (POBA) was introduced in the 1980 s, but is of limited use today due to high restenosis rates (30–50 %) and possible complications of acute and subacute vessel closure [1]. Thus, bare metal stents (BMS) were developed to overcome these limitations at the expense of in-stent restenosis due to intimal hyperplasia. Newer generation drug-eluting stents (DES) proved to effectively suppress neointimal proliferation and delayed restenosis rates are now only 5–15 % [2]. However, a foreign body and local hypersensitivity reactions seem to play a pivotal role for the development of restenosis and late stent thrombosis after stenting [3, 4]. Also, positive vessel remodeling is hindered by a persistent metallic cage and preserving vasomotion after angioplasty is of growing interest.

So far, DCB angioplasty has proven its value in the interventional treatment of de novo stenosis in small coronary vessels, in-stent restenosis or for angioplasty of the side branch in bifurcation stenosis [5–14]. However, there is still limited data and reluctance to treat stenoses in native coronaries of any size with this interventional strategy due to the fear of periprocedural and subacute complications with unfavorable outcome and potential need for bail-out stenting. DCB angioplasty requires only 4 weeks of dual antiplatelet therapy (DAPT), which is a considerable advantage since bleeding complications after PCI have a negative impact on clinical outcomes, and are of health-economical interest.

## Aims of the study

 We aimed to investigate the feasibility of fractional flow reserve (FFR)-guided use of paclitaxel-coated balloons (PCB) with only provisional bare metal stenting for elective PCI of de novo coronary lesions in an all-comers population. Outcomes were evaluated at 6 months by invasive follow-up (f/u) using angiography and optical coherence tomography (OCT) in addition to clinical data at 1 year f/u.

## Methods

### Trial design and study setting

The presented study (“Optical coherence tomography to investigate FFR-guided DCB-only elective coronary angioplasty—OCTOPUS-2”; http://www.clinicaltrials.gov: NCT02120859) is a prospective, single-center, single-arm, investigator-initiated study that was conducted between 10/2012 and 11/2014 at the University Hospital of Jena, Germany. The study was approved by the local ethical committee and conducted according to the principles of the Declaration of Helsinki. All study participants gave informed written consent. The predefined sample size was 50 patients.

### Interventions

**Fig. 1:**
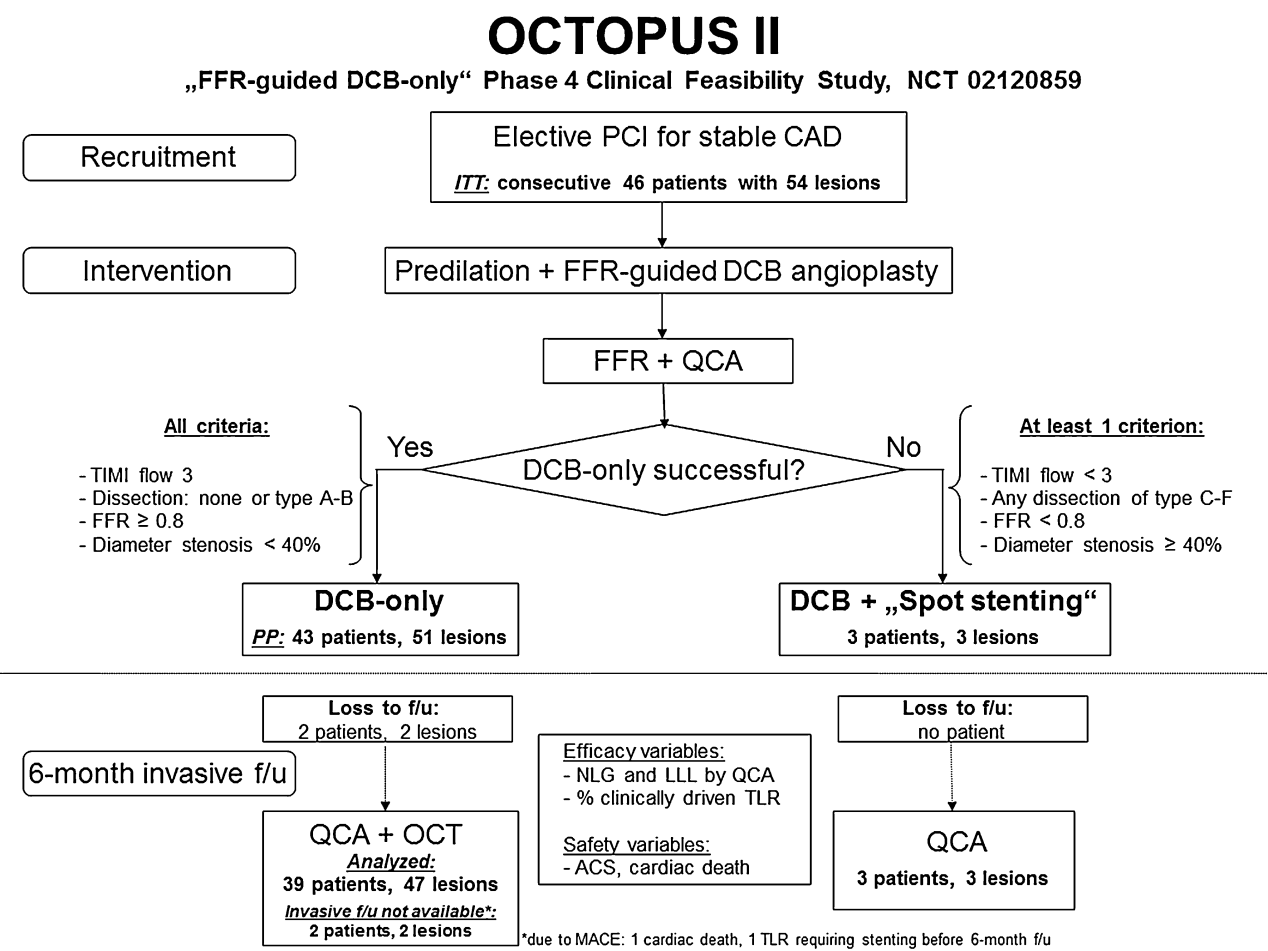
Patient flow chart of the different study phases

Patients with stable coronary artery disease and indication for elective PCI of a de novo stenosis were suitable for study participation (Online Resource, Fig. 1). Quantitative coronary angiography (QCA) and FFR using an intracoronary bolus of adenosine (60 µg for the right coronary artery and 120 µg for the left coronary artery) after placement of a pressure wire far distal from the stenosis were performed at baseline. FFR was not performed in diameter stenosis <40 %, or >75 % in patients with conclusive symptoms and pathologic non-invasive ischemia testing. If FFR at baseline >0.8, PCI was deferred, otherwise predilation with a non-coated semi-compliant balloon was performed. Provisional stenting was only performed in case of residual diameter stenosis ≥40 % or flow-limiting dissections (Fig. 1). Otherwise, the lesion was dilated with a Sequent Please^®^ paclitaxel-coated balloon (B Braun Melsungen GmbH, Germany). Slow balloon inflation, longer inflation time (60 s), low inflation pressures and higher balloon/artery (b/a) ratio were attempted to avoid large dissections and to achieve sufficient angioplasty results.

### Outcome measures

Invasive f/u with QCA and OCT was attempted in all patients after 6 months. Outcome measures were predefined.

1. Primary outcome measures
*Late lumen loss* [LLL in mm; minimal luminal diameter (MLD)_postprocedural_ − MLD_6-month f/u_] within the treated segment by QCA
2. Secondary outcome measures
*Net luminal gain* (NLG in mm; MLD_6-month f/u_ − MLD_baseline_)
3. OCT-derived outcomes
  Lumen- and vessel-size measurements, plaque evaluation and dissection healing within the treated segment at 6 months
4. Clinical outcomes
  Target lesion revascularization (TLR)
  Major adverse cardiovascular events (MACE), defined as cardiac death, acute myocardial infarction, and revascularization (target and non-target vessel revascularization) at 6-month and 1 year clinical f/u. Clinical f/u was conducted by a structured telephone interview and using the hospital database.

### Data collection

**Fig. 2:**
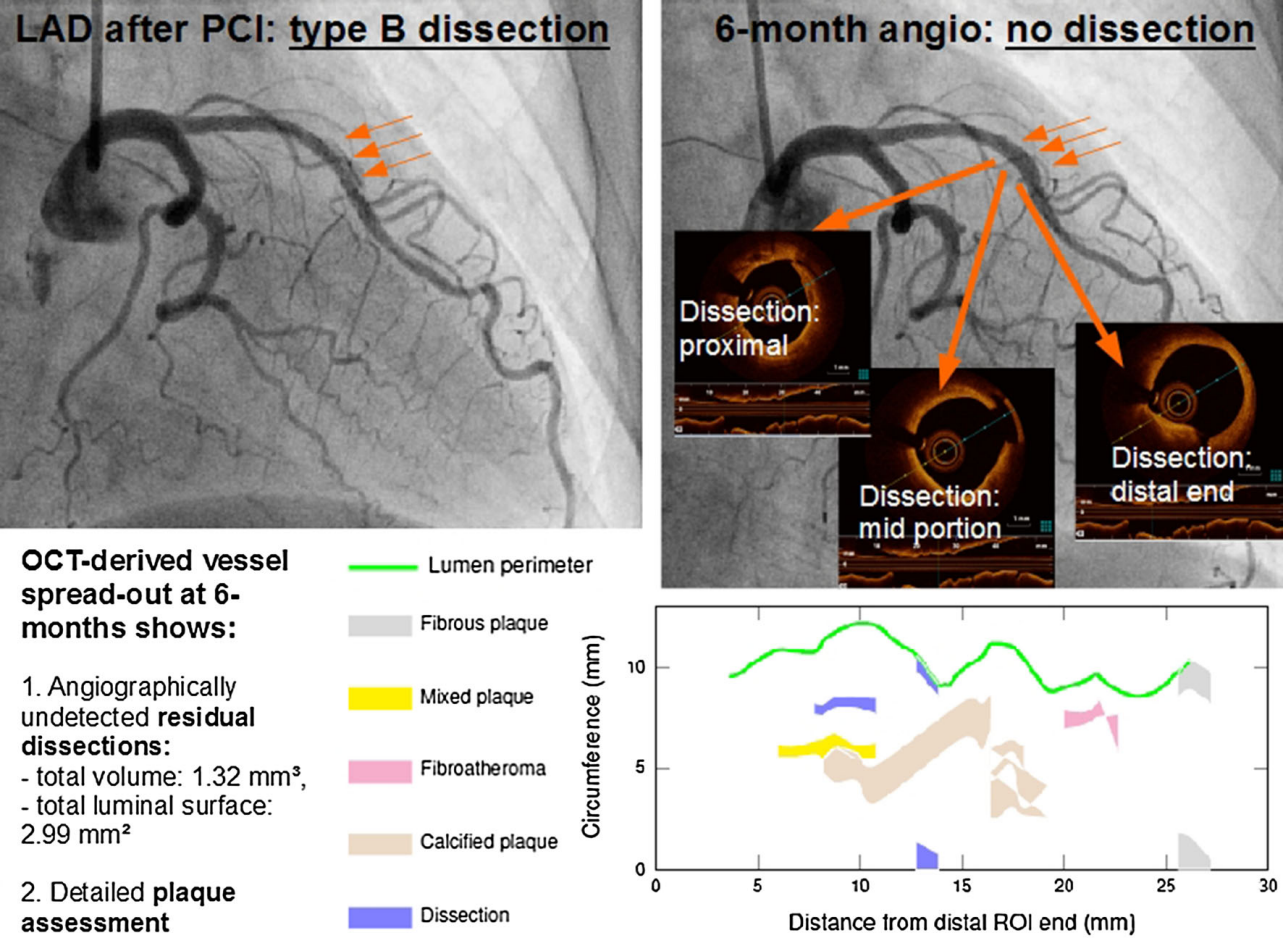
Example of a performed DCB-only PCI of the left anterior descending (LAD) artery with final PCI result showing an angiographically determined type B dissection (*left*) with complete resolution during re-angiography after 6 months (*right*-*top*). However, OCT imaging at 6-months f/u still reveals some minor dissections, which are illustrated with coronary plaque measurements in a spread-out vessel chart of the treated vessel segment (*right*-*bottom*)

QCA according to the 15-coronary tree segment system was assessed offline by two independent observers (C.D., S.O.) using the same projections for baseline and f/u (CAAS version 5.9.2, 2012, Pie Medical Imaging, Maastricht, Netherlands). Dissections were classified as type *A*–*F* according to the National Heart Lung and Blood Institute (NHLBI) criteria [15]. Distal coronary flow was assessed according to thrombolysis in myocardial infarction (TIMI) flow classification [16]. OCT images were acquired with an automated pullback of 20 mm/s (St. Jude Medical Ilumien™), analyzed offline and blinded to the coronary angiogram. A computational algorithm was applied for dissection and plaque analysis as described before: Plaque morphology was assessed according to the international consensus [17, 18]. Plaques and dissections were manually traced in each cross section, and according volumes were computed through the integral of cross-sectional measurements. Luminal surface areas were calculated by applying the Shoelace formula. Calculated OCT parameters were displayed as spread-out vessel charts (Fig. 2).

### Statistical analysis

Patient data were archived in a customized Microsoft Access (Microsoft Inc., Redmond) database. SPSS (version 21, IBM SPSS statistics) was used for statistical analysis. Continuous and normally distributed variables were analyzed with the Student *t* test and categorical variables with the Pearson *χ* ^2^ test. Two sided *p* values <0.05 were accepted as statistically significant. Paired *t* test and Bland and Altman agreement analysis were used for comparison of QCA and OCT measurements.

## Results

### Study population

We included 46 consecutive patients with 54 study lesions (Fig. 1). Provisional stenting was necessary in three study patients (5.9 %; three lesions). Two patients (two lesions) were lost to 6-month invasive f/u (loss of f/u 4.3 %) due to deterioration of renal function in one patient and refusal of re-angiography in another patient. However, clinical f/u data are available for all patients. Six-month invasive f/u was completed in 39 patients (47 lesions) including 45 lesions with sufficient OCT imaging quality (Fig. 1).

### Baseline clinical and procedural characteristics

Tables 1 and 2 show baseline clinical characteristics and procedural data of the study population. Acute lumen gain (MLD_postprocedural_ − MLD_baseline_) was 0.98 ± 0.31 mm. Mean FFR-raised from 0.64 ± 0.19 to 0.91 ± 0.06 (*p* < 0.05, Table 2). To achieve a sufficient increase of FFR relatively high b/a ratios (oversize ratio 1.27 ± 0.19) were applied (Table 2). Within 24 h after PCI troponin I elevations were detected in three patients with a mean value of 213.3 ± 141.9 ng/ml. However, ECG changes were not observed and clinical course remained uneventful in all patients without further actions needed.

**Table 1:** Baseline clinical characteristics of the study population, intention-to-treat analysis of 46 patients

	Characteristics	*n* = 46 patients
		Mean ± SD or (%)
Demographics	Age	67.1 ± 10.6
	Male gender	29 (63 %)
Coronary artery disease	Previous myocardial infarction	22 (47.8 %)
	3-vessel disease	18 (39.1 %)
	Previous CABG	8 (17.4 %)
Diabetes	Diabetes mellitus type 2	18 (39.1 %)
	HBA1c (%)	6.97 ± 0.38
Dyslipidemia	Suboptimally controlled	14 (30.4 %)
	LDL (mg/l)	107.9 ± 40.2
	HDL (mg/l)	42.9 ± 11.6
Risk profile	Cigarette smoking	17 (37 %)
	Hypertension	40 (87.0 %)
	Peripheral artery disease	7 (15.2 %)
	Previous stroke	1 (2.2 %)
	Family history	3 (6.5 %)
Renal function	Serum creatinine (mg/dl)	1.13 ± 0.6
	Glomerular filtration rate (ml/min)	69 ± 21.2

*SD* standard deviation, *CABG* coronary artery bypass grafting, *HBA1c* hemoglobin a1c, *LDL* low-density lipoprotein, *HDL* high-density lipoprotein

**Table 2:** Procedural and lesion characteristics and 6-month invasive follow-up, per-protocol analysis

	Characteristics	Baseline	PCI results	6-month F/U
		*N* = 51 lesions	*N* = 51 lesions	*N* = 47 lesions
		Mean ± SD or *N* (%)	Mean ± SD or *N* (%)	Mean ± SD or *N* (%)
Target lesion	LAD/LCX/RCA	19/21/11		
	Bifurcation lesion	9 (17.6 %)		
	AHA/ACC lesion type A/B/C	6/31/14		
QCA	Reference lumen diameter (mm)	2.32 ± 0.48	2.52 ± 0.44*	2.27 ± 0.0.87°
	Minimal lumen diameter (mm)	0.82 ± 0.26	1.80 ± 0.42*	1.85 ± 0.73*
	Diameter stenosis (%)	63.9 ± 10.9	28.1 ± 10.8*	19.5 ± 20.4*°
	Lesion length (mm)	15.9 ± 5.5		
Intervention	FFR	0.64 ± 0.19	0.91 ± 0.06*	
	DCB diameter (mm)	2.84 ± 0.34		
	DCB length (mm)	24.22 ± 5.86		
	DCB dilation time (s)	53 ± 10		
	DCB pressure (bar)	10.8 ± 2.3		
	DCB/artery ratio	1.27 ± 0.19		
	Fluoroscopy time (min)	9.9 ± 7.4		
	Dissections in angiography	0 (0 %)	27 (52.9 %)*	4 (7.8 %)*°
	Troponin elevations	0 (0 %)	3 (6 %)	0

*p* < 0.001: * vs. baseline, *p* < 0.05: ° vs. PCI result

*LAD* left anterior descending artery, *LCX* left circumflex, *RCA* right coronary artery, *SD* standard deviation, *N* number, *FFR* fractional flow reserve, *DCB* drug-coated balloon, *DCB/artery ratio* nominal balloon diameter/RLD-1, *QCA* quantitative coronary angiography

### Clinical outcomes and adverse events

Three major adverse cardiovascular events were observed in the study population within 6 months (Intention-to-treat analysis, Table 3). There were two target lesion failures with the need for revascularization: one repeated PCI due to a progressive dissection was necessary within 4 weeks after DCB-only angioplasty and one coronary artery bypass graft was done due to significant in-stent stenosis after DCB use with provisional bare metal stenting. One cardiac death due to ventricular fibrillation occured, however, this was unrelated to the study procedure, since autopsy revealed a patent target vessel.

**Table 3:** Primary, secondary, and clinical outcome measures at follow-up

	Outcome measures	*N* = 39 patients (47 lesions)
Primary	LLL (mm)	−0.13 ± 0.44
Secondary	NLG (mm)	1.1 ± 0.53
Clinical	TLR at 6-month f/u	1/2^a^
	MACE at 6-month f/u	2/3^a^
	MACE at 1-year f/u	2/3^a^

Results are shown as per-protocol analysis

*LLL* late lumen loss, *NLG* net luminal gain, *TLR* target lesion revascularization, *MACE* major adverse cardiovascular events, *f/u* follow-up

^a^Intention-to-treat analysis (*N* = 54 lesions in 46 patients)

### Invasive evaluation at 6-month follow-up

#### Angiography

Our results show a trend toward progressive lumen enlargement at the stenotic site and positive net lumen gain at 6-month f/u (Table 3; Online Resource: Figs. 3a–c). More importantly, no focal aneurysm formation or restenosis at 6-month f/u after DCB-only angioplasty were observed. Most of the initially observed type A and B dissections (*N* = 27 of 51 lesions; 52.9 %; Table 2) were completely healed at f/u (Fig. 2). Angiographically only four small type A dissections were found at 6-month f/u without need for further actions.

#### Oct

OCT imaging at 6-month f/u was focused on dissection assessment (Table 4). OCT revealed small focal dissections in 42 % of the lesions that were judged angiographically as insignificant. Contrary, in two out of four angiographical type A dissections at f/u, OCT found no evidence of focal dissection (Table 4; Fig. 2). We found no predictors (e.g., vessel angulation, vessel diameter, lesion length, calcification, number of dilation, oversize ratio, diabetes, renal insufficiency, LDL-levels, etc.) for the occurrence of dissections either after PCI or at 6-month f/u.

**Table 4:** Analysis of local dissections of the treated vessel segment post-procedural and at 6-month invasive f/u with angiography and OCT (*N* = 45 lesions with sufficient OCT imaging quality and matching angiography)

Dissection assessment by OCT at f/u	Angiography after PCI			Angiography at f/u		
	No dissection (*N* = 19)	Type A dissection (*N* = 19)	Type B dissection (*N* = 7)	All dissections (*N* = 26)	No dissection (*N* = 41)	Type A dissection (*N* = 4)
Dissections present at f/u	8 (42 %)	10 (53 %)	2 (29 %)	12 (46 %)	18 (42 %)	2 (50 %)
Total dissection length (mm)	0.2 ± 0.42	0.17 ± 0.32	0.17 ± 0.37	0.17 ± 0.2	0.19 ± 0.37	0.07 ± 0.11
Total dissection volume (mm^3^)	0.04 ± 0.08	0.03 ± 0.07	0.21 ± 0.49	0.06 ± 0.18	0.07 ± 0.22	0.01 ± 0.03
Total luminal surface area (mm^2^)	1.57 ± 4.67	1.25 ± 4.22	0.5 ± 1.11	1.05 ± 3.64	1.36 ± 4.24	0.38 ± 0.67
Peak dissection depth (mm)	0.18 ± 0.33	0.24 ± 0.34	0.17 ± 0.32	0.22 ± 0.32	0.2 ± 0.32	0.16 ± 0.28

All values are given as mean ± SD

*OCT* optical coherence tomography, *PCI* percutaneous coronary intervention

OCT plaque analysis revealed 4.1 ± 3.0 plaques with a total plaque volume of 21.8 ± 24.6 mm^3^ and a total luminal plaque surface area of 10.8 ± 9.4 mm^2^ per investigated target vessel segment. In detail, there were 0.2 ± 0.5 fibrotic plaques, 1.3 ± 1.6 calcified plaques, 1.5 ± 1.3 mixed plaques, and 1.1 ± 1.2 fibroatheromas per vessel segment (Fig. 2). Thin-cap (< 65 µm) fibroatheromas (TCFA) were not observed. No association between plaque burden and the incidence of dissections was found.

There was a good correlation (*r* = 0.738; 95 % CI −0.37 to −0.11 mm; *p* < 0.001) between MLD measurements at f/u with QCA (MLD_QCA_ 1.97 ± 0.62 mm) and OCT (MLD_OCT_ 1.73 ± 0.44 mm). Agreement between QCA and OCT according to Bland–Altman data plotting was higher in smaller diameter vessels.

## Discussion

The main findings of this prospective study are: (1) DCB-only angioplasty in all-comers is effective and safe with no subacute vessel closure and low MACE rate (4.7 % at 6-months) with only 4 weeks of DAPT, (2) a trend toward positive vessel remodeling with late lumen enlargement is seen at 6-month invasive f/u, (3) conversion rate to bail-out stenting under FFR guidance is very low (6 %), (4) clinically silent type A and B dissections are mostly healed at f/u, (5) angiography is unreliable for a correct diagnosis of small residual dissections.

Evidence for DCB-only angioplasty as a primary interventional strategy is growing. So far, published data are stemming from mainly retrospective registries and case reports [14, 19–22]. One serial, prospective study using IVUS and FFR analyzed only 27 from 48 initially treated patients [23]. To the best of our knowledge, this is the first prospective, officially registered study with a dedicated, predefined procedural protocol that investigates the feasibility of elective DCB-only PCI in all-comers and provides a nearly completed 6-month invasive and clinical f/u in all patients. Earlier studies of DCB-only angioplasty of de novo stenoses showed a high conversion rate to stenting (11.9–32.8 %), or investigated a preselected study population [20, 21, 23]. Contrary, bail-out stenting in our study was necessary only in 6 % of cases.

DCB angioplasty leads to lumen enlargement at 6-month invasive f/u in many patients expressed as overall LLL of −0.13 ± 0.44 mm (Online Resource: Figs. 3a–c). Our results on positive vessel remodeling are in agreement with previous studies also showing luminal gain at variable f/u times (Scheller 2013: LLL −0.25 to −0.18 mm; Kleber 2015: late lumen increase in 69 % of the lesion, Her 2016: LLL: −0.12 ± 0.30 mm; Shin 2015: LLL 0.05 ± 0.27 mm; Ann 2016: LLL 0.02 ± 0.27 mm) [14, 19, 21–23]. Only the Valentine´s II trial showed a LLL of 0.38 ± 0.39 mm at 6–9 months f/u. However, this might underline the necessity of adequate lesion preparation since predilation in the Valentine´s II trial was conducted in only 85 %, and DCB diameters were significantly smaller despite comparable RLDs. Contrary to the consensus on DCB treatment, the mean *b*/*a* ratio of 1.27 in this study was considerably higher than recommended (0.8–1.0), but is in line with values reported by other studies (1.15–1.18). Thus, oversizing seems of crucial importance to achieve sufficient acute and long-term results [14, 21, 22].

Post-interventional FFR is less well evaluated, but pressure gradient measurements proved to be a useful and reliable tool to guide immediate results of angioplasty [24, 25]. In our study, we used a cut-off of >0.8 for post-interventional FFR, which one might find unusually low to accept final angioplasty results. FFR values <0.95 post-stenting are associated with adverse long-term results [24]. However, to the best of our knowledge, cut-off values for FFR after a DCB-only procedure have never been investigated yet. Also, given the higher acute lumen gain after stent implantation, and the expected late lumen enlargement after DCB-only angioplasty, one must not extrapolate data from publications investigating stent results. In this light, clinical and angiographic data in our study seem reassuring for FFR values > 0.8 after DCB-only, but this hypothesis needs confirmation in further studies with longer follow-up times.

Polymers in DES platforms are associated with disadvantageous vessel wall effects, while DCB are polymer-free. DCB angioplasty allows uniform distribution of the antiproliferative drug to the vessel wall, and significantly higher paclitaxel-concentrations are used compared to paclitaxel-eluting stents [26, 27]. However, properties of DCB´s should not be understood as “class effects” [28]. Current evidence for vessel wall expansion is the strongest for paclitaxel and is histologically proven as media wall thinning and cell necrosis in addition to inhibition of smooth muscle cells [18, 29–31]. Out of the available paclitaxel-coated balloons, the Sequent Please™ DCB used in this study has been best investigated. This is of importance since vessel wall effects of paclitaxel are known to be dose-dependent [32, 33].

We found no aneurysm formation at the stenotic site at f/u, which confirms another study that specifically investigated the incidence of focal coronary artery aneurysm after DCB angioplasty [34].

Plaque regression might also account for the specific effects of positive vessel remodeling. A small, serial IVUS study found no change of mean plaque area 9 months after DCB angioplasty but a significant decrease of atheroma volume [23]. The mentioned study also found plaque modulation since out of nine initially observed TCFA’s only five TCFA’s were seen at f/u, which the authors interpreted as a result of cap thickening and transformation to pathological intimal thickening [23]. In line with this observation, we did not find any TCFA’s at 6-month f/u within the treated segment using OCT, which is more reliable in determining TCFAs than IVUS [35].

Another focus of our study was the evaluation of dissections as a result of angioplasty and its impact on midterm outcomes. By applying the mentioned procedural precautions and intracoronary pressure monitoring, bail-out stenting was grossly preventable. From the only small residual dissections seen at baseline, most dissections were completely healed at f/u, which is in line with a previously published study [36]. Interestingly, angiography is largely misleading in evaluation of small dissections of NHLBI type A, and no real association between dimensions of dissection investigated by OCT and angiography was found.

Novel interventional approaches for the therapy of stable stenosis try to eliminate the necessity for a permanent foreign body since it has been shown that in-stent restenosis is at least partly caused by local hypersensitivity reactions and neoatherosclerosis in response to the metal material, the polymer or the antiproliferative drug [37]. Lately, bioresorbable vascular scaffolds (BVS) are in the focus of interest. Interestingly, for both “metallic free” concepts of BVS and DCB-only angioplasty, late lumen enlargement has been shown, which is contrary to the well-known vessel response after POBA, BMS or DES implantation resulting always in late lumen loss [38, 39]. Also, restoration of vasomotion after angioplasty is advantageous and might maintain positive long-term outcomes including relief of angina symptoms [39, 40]. However, prolonged DAPT for 12 months is needed after BVS implantation. Also, rates of acute and late scaffold thrombosis are not negligible, and seem to be higher compared to newer generation DES [41]. Degradation of the ABSORB™ BVS, which is currently the best investigated scaffold, is with >2 years rather slow and the optimal degradation rate of vascular scaffolds remains to be determined [39]. Contrarily, duration of DAPT after DCB-only angioplasty is with 4 weeks only minimal and reports on acute vessel closure after PCI are very rare. Thus, DCB angioplasty as a stand-alone procedure might be a promising trade-off between POBA, DES, and BVS. On the other hand, it has been shown that BVS has the capacity to “seal” underlying coronary plaques [39, 42]. Whether or not this translates into a favorable long-term course remains nevertheless unclear. Changes in coronary plaques after a DCB-only procedure need to be further investigated, before definitive conclusions and clinical recommendations comparing both concepts can be formulated.

### Clinical implications

FFR-guided DCB-only PCI using the Sequent Please balloon is feasible, safe, and effective irrespective of vessel size. Small, non-flow-limiting dissections type A and B with normal FFR values >0.80 do not require stenting and show favorable clinical outcomes. Operator’s training including lesion preparation, choice of balloon size, inflation time and pressure, and evaluation of dissections is necessary to successfully apply a DCB-only strategy as stand-alone procedure and to achieve good clinical outcomes.

### Limitations

This study has a relatively small sample size and was intended as a feasibility study to prove the concept of an elective FFR-guided DCB-only angioplasty. Thus, our study is not a randomized trial comparing DCB-only PCI to another study intervention. Since we did not perform OCT at baseline, plaque regression and dissection healing could not be recorded and further serial OCT studies specifically focusing on this aspect are needed.

## Conclusion

The concept of FFR-guided DCB-only angioplasty without stenting of de novo lesions in stable CAD is effective and safe with post-procedural DAPT duration of only 4 weeks. Local delivery of paclitaxel induces persistent vessel remodeling leading to lumen enlargement in many cases at 6-month f/u. Intracoronary imaging and FFR are useful to guide angioplasty and to avoid unnecessary stenting. Larger studies are needed to redefine the management of non-flow-limiting dissections and to evaluate long-term outcomes after DCB-only angioplasty in comparison to implantation of drug-eluting stents and scaffolds.

## Electronic supplementary material

Below is the link to the electronic supplementary material.

Supplementary material 1 (DOCX 87 kb)
