# Supplementary material for: Fractional flow reserve-guided coronary angioplasty using paclitaxel-coated balloons without stent implantation: feasibility, safety and 6-month results by angiography and optical coherence tomography
Source: Clin Res Cardiol. 2016 Jul 5;106(1):18–27. doi: 10.1007/s00392-016-1019-4 (PMC5226992; doi:10.1007/s00392-016-1019-4)
Supplement: Supplementary file 1 — Supplementary material 1 (DOCX 87 kb) [file 392_2016_1019_MOESM1_ESM.docx]

**SUPPLEMENT**

**Eligibility Criteria**

Inclusion Criteria

- indication for elective PCI according to the guidelines of European Society of Cardiology, American Heart Association/American College of Cardiology
- Age > 18 years
- written consent
- Native de novo coronary lesion suitable for angioplasty and OCT imaging

Exclusion Criteria

General

- Pregnancy and breast feeding mother
- Co-morbidity with an estimated life expectancy of < 50 % at 1 year
- Scheduled major surgery in the next 6 months
- Not able to give informed written consent or non-compliance
- Participation in other PCI trial
- Acute coronary syndromes and cardiogenic shock
- Known allergy to aspirin, thienopyridines or against taxol derivates

Lesion related

- Culprit lesion within the proximal 10 mm of the right or left coronary artery
- previous revascularization of the possible study lesion
- vessel supplied by an aortocoronary bypass
- estimated lesion length > 30 mm
- reference luminal diameter > 4 mm

**Figures 3 a-c:** Comparison of (a) minimal and reference lumen diameter (MLD, RLD), (b) % diameter

stenosis, and (c) cumulative distribution percentages of minimal lumen diameter (MLD) at

baseline, postprocedural and at 6-month f/u

**Figure 3a**


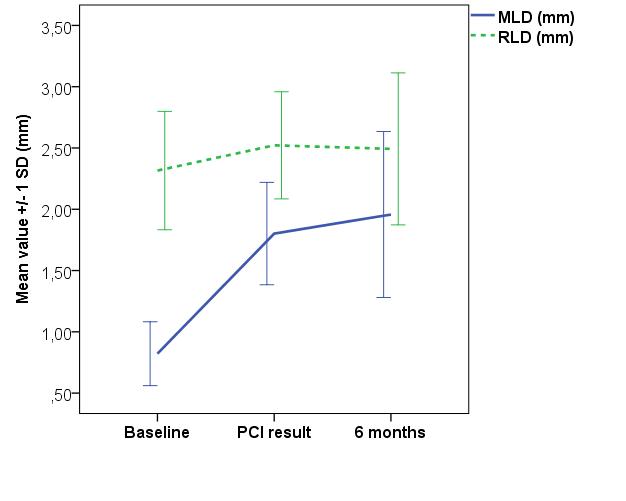


**Figure 3b**


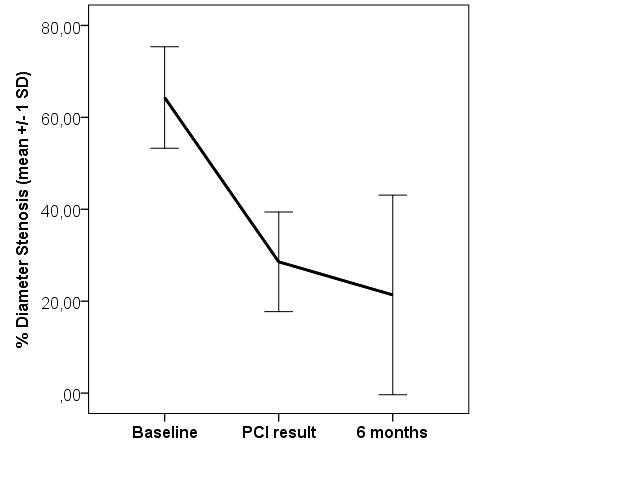


**Figure 3c**

**
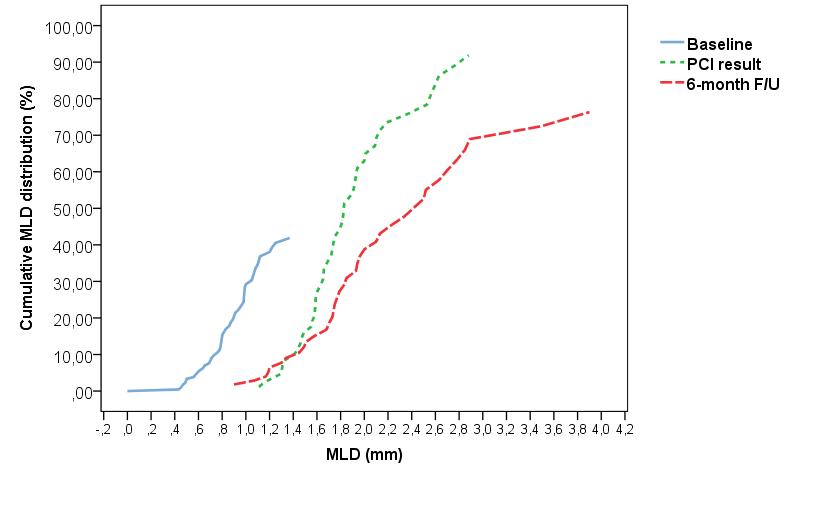
**
